# Comparison of transrectal and transabdominal transducers for use in fast localized abdominal sonography of horses presenting with colic

**DOI:** 10.3389/fvets.2023.1307938

**Published:** 2024-01-04

**Authors:** Hanna Haardt, Alfredo E. Romero, Søren R. Boysen, Jean-Yin Tan

**Affiliations:** ^1^Faculty of Veterinary Medicine, University of Calgary, Calgary, AB, Canada; ^2^Department of Large Animal Surgery, Anaesthesia and Orthopaedics, Ghent University, Merelbeke, Belgium

**Keywords:** ultrasound, probe, abdomen, diagnostic imaging, rectal

## Abstract

Abdominal ultrasonography is valuable in the diagnosis of equine colic. Fast localized abdominal sonography of horses (FLASH) enables practitioners with limited experience to perform ultrasonography in emergency settings. However, many practitioners only possess rectal format linear array transducers (RFLT). The hypotheses are: (a) A low frequency curvilinear transducer (LFCT) and RFLT will detect free abdominal fluid and abnormal small intestinal loops with similar frequency during FLASH, and (b) there will be a difference between the transducers for detection of gastric abnormalities and nephrosplenic entrapment. The objective is to compare transcutaneous abdominal ultrasonographic detection of abnormalities in horses presenting with colic using a LFCT and RFLT. Twenty-four horses requiring FLASH for investigation of colic were enrolled. Horses that were too painful to undergo transcutaneous abdominal ultrasonographic examination were excluded. A single investigator performed FLASH on all horses using a RFLT, while one of three other clinicians simultaneously performed FLASH using a LFCT. Comparison of abnormal findings between the two transducers was performed using Chi square, Fisher’s exact or Wilcoxon tests. The incidence of identification of abnormal findings was similar between the two transducers for all comparisons except the visibility of the left kidney and stomach (kidney LFCT 81.25% vs. RFLT 22.92%, stomach LFCT 87.5% vs. RFLT 62.5%). While there are limitations to using a RFLT to identify nephrosplenic entrapment of the colon and detection of the stomach, it reliably detects other common abnormalities, including peritoneal effusion, lesions of the small intestine, and changes to the wall of the large colon and cecum.

## 1 Introduction

Abdominal ultrasonography is an important diagnostic modality in the assessment of the equine colic patient (1–3). Due to technical advancements ultrasound image quality has improved throughout the years and ultrasonography has become an integral part of a colic workup in many clinics and practices (4–7). Traditionally ultrasonographic examination of the abdomen is performed using a 2–5 MHz low frequency curvilinear transducer (abdominal transducer) (2, 8). While first-opinion equine veterinarians commonly possess 5–10 MHz rectal format linear array transducers (transrectal transducer), only 8% of ultrasound machines purchased in equine configurations in Canada over the last 5 years from an international company include an abdominal transducer.^1^ In a previous study, the authors established that a transrectal transducer produces ultrasonographic images of sufficient quality to allow incidence of organ identification similar to that of an abdominal transducer except for the left kidney, left liver, and stomach, when performing transcutaneous equine abdominal ultrasonography (9). The difference in detection of abnormalities between transducers was believed to be due to the depth limitation of the transrectal transducer compared to the abdominal transducer (the transrectal transducer used in that study operated at 8–13 MHz, and the abdominal transducer at 3.0–3.8 MHz) (9). A thick haircoat and horses with increased abdominal fat were discussed as possible factors influencing the ability to detect abdominal structures, but this data was not specifically collected and assessed in the previous study.

Ultrasonographic protocols, such as fast localized abdominal sonography of horses (FLASH), have proven to be effective in the emergency diagnosis of major abnormalities causing colic (6). Expedited diagnosis of key abnormalities such as excessive peritoneal effusion and small intestinal and colon pathology may ensure immediate referral for intensive medical or surgical intervention.

The objective of this prospective clinical study was to compare ultrasonographic detection of abnormalities between an abdominal and transrectal transducer in horses presenting for colic, using a transcutaneous abdominal scanning technique. A second objective was to determine if hair coat and body condition score (10) would influence ultrasonographic findings. We hypothesize that both transrectal and abdominal transducers will detect free abdominal fluid and abnormalities of small intestinal loops, such as distention, absent or decreased motility, and thickened wall, with similar frequency. We also hypothesize that there will be a difference between the transducers for detection of gastric conditions such as dilation, thickening of the wall, or masses, and left dorsal displacement of the colon.

## 2 Materials and methods

Animal ethics approval was obtained from University of Calgary Animal Care Committee (AC19-0116) and informed consent was obtained. Horses greater than 1 year of age that presented to a referral clinic^2^ for colic and had transcutaneous abdominal ultrasonography requested by the attending clinician were enrolled. Horses clinically assessed as being in too much discomfort to complete the ultrasonographic examination (unable to stand in stocks despite sedation), and cases where the owner declined ultrasonography due to financial restrictions, were excluded from the study.

Based on availability during the study period of 2 months (December 1 2019 to January 31 2020) 24 horses were enrolled. Breed, body condition score, sex, body weight, and length of the hair coat was recorded at the time of presentation. Working diagnosis (final diagnosis if confirmed through surgery or post-mortem examination) and duration of the ultrasonographic examination were recorded. Horses were restrained in stocks for the duration of the ultrasonographic exam. Due to the time sensitivity of the procedure in evaluating colic emergencies, the haircoat was not clipped and 70% isopropyl alcohol was used as the coupling agent. Horses that were excessively soiled were brushed prior to performing ultrasound. Portable veterinary ultrasound machines were used (Mindray M5 vet, Shenzhen Mindray Bio-Medical Electronics Co., Ltd., Shen Zhen, China). The transrectal (linear) transducer was a 5–8 MHz frequency transducer (6LE5Vs Shenzhen Mindray Bio-Medical Electronics Co., Ltd., Shen Zhen, China) with a maximum depth setting of 30 cm, however, no image could be obtained beyond a depth of 12 cm at the lowest frequency in B-Mode (5 MHz). An initial depth setting of 12 cm and a frequency of 5 MHz was used for all examinations. These values were then adjusted based on operator preference during the examination. Using FLASH 7 areas were evaluated on each horse: Ventral abdomen, left cranial abdomen, caudal dorsal and ventral abdomen (left and right) and the right thorax (6). The standard abdominal transducer was a convex transducer of 2.5–6 MHz (3C5s Shenzhen Mindray Bio-Medical Electronics Co., Ltd., Shen Zhen, China). The initial settings for the abdominal transducer were 25 cm depth and 2.5 MHz, which were adjusted based on operator preference.

After the attending clinician completed the presenting physical examination, horses were placed in stocks. The working diagnosis was determined by the attending clinician based on clinical, rectal, and ultrasonographic examination and recorded following completion of the ultrasound examination. Horses were simultaneously examined bilaterally by two out of the four participating examiners, whom were veterinarians that had between 6 months and 5 years of clinical experience. All examiners involved in the study received standardized ultrasonographic training consisting of one full day of practical training in abdominal ultrasound and a 2-h theoretical lecture-based presentation. All examiners had equal experience with abdominal ultrasound. The examiner operating the abdominal transducer decided the side of the horse that they commenced the examination based on the location of the suspected clinically relevant lesion, while the examiner with the transrectal probe started on the opposite side of the abdomen. The same veterinarian (H.H.) with 1.5 years of experience completed all ultrasonographic examinations with the transrectal transducer, whereas one of three additional examiners performed the simultaneous ultrasonographic examination using the abdominal transducer. Each examiner completed a FLASH, recording results on a standardized FLASH score sheet (6). When the examiner using the abdominal transducer signaled completion of the examination on their side of the abdomen, the examination with the transrectal transducer was interrupted, irrespective of whether it had been completed or not. This was done to avoid any negative impact on patients enrolled in the study. The time taken to complete each exam, or time to interruption of the transrectal transducer exam, was recorded. At least one cine-loop of 5 s was recorded at each of the 7 sites included in the FLASH. Each examiner independently completed the FLASH score sheet. Cine-loops were redacted and randomized for blinding and retrospectively analyzed by two specialists 1 board-certified large animal internal medicine specialist with 14 years of experience [J.T., DVM, DACVIM (LAIM)], and 1 board-certified large animal surgeon with 14 years of experience [A.R., DVM, DACVS (LA)]. Upon review of the recorded Cine-loops, the experts completed a modified FLASH scoring sheet. Abnormalities were categorized according to the FLASH score sheets. The data of all score sheets was pooled for statistical analysis. Further treatment of the horses was at the discretion of the attending clinician, and included withholding food, intravenous or nasogastric fluids, laxatives, jogging, and or surgery.

All data was tested for normality using a Shapiro–Wilk test, and subsequently analyzed using parametric or non-parametric tests. A *p* value <0.05 was considered statistically significant. To determine any statistically significant difference between transducers for dichotomous data a Chi square test (when *n* ≥ 10 in all cells of a 2 × 2 table) or Fisher’s Exact Test (*n* ≤ 5 in any cell of a 2 × 2 table) was used. To determine any difference in the mean (or median) between groups, a paired t-test or Wilcoxon matched-pairs signed rank test was used for data that passed or failed normalcy, respectively. A *t*-test or Mann Whitney test was used to compare differences between transducers for continuous variables that passed or failed normalcy, respectively. For haircoat length, numerical values from 1 to 4 were assigned with 1 designated as a clipped haircoat, 2 a light coat (horse was wearing a heavy rug or had been stabled indoors), 3 a winter coat, and 4 a heavy winter coat. Abnormal motility of the small intestine was defined as absence or reduction of peristaltic waves. Wall thickness was considered increased when it exceeded 0.3 cm for small intestine and 0.5 cm for colon. Graphpad prism (version 8.4.2, San Diego, CA, United States) was used to perform all statistical analysis. Mean and median age, body weight, and BCS (10) were calculated using Excel (Microsoft Office Professional 2019, Redmont, Washington, United States).

## 3 Results

Twenty-four horses of various breeds (10 Warmblood, 8 Quarter Horse, 4 Thoroughbred, 1 Fjord horse, and 1 Percheron cross) and sex (15 geldings, 8 mares, and 1 stallion) were enrolled between December 2019 and January 2020. Age ranged from 3 to 27 years (median 12), weight from 402 kg to 610 kg (median 500 kg), and the body condition score from 2 to 8 (median 6). A presumptive diagnosis was available in 22/24 horses: 5 horses presented with right dorsal displacement of the colon, 5 with primary impaction of the large colon with ingesta, 4 horses with left dorsal displacement of the colon out of which one was a nephrosplenic entrapment, 5 horses with tympany of the cecum or colon, 2 horses with colitis and 1 horse with a suspected gastroduodenojejunitis. Diagnoses were made by the attending clinician based on clinical examination including physical examination, bloodwork, rectal palpation, and ultrasonography. All patients were medically managed, thus there is no final diagnosis available. For two horses the authors could obtain a final diagnosis: one horse that had a 720-degree torsion of the colon, which underwent a ventral midline laparotomy and a pedunculated, strangulating, lipoma that was diagnosed on post-mortem examination in one horse that was euthanized. All other horses responded to medical management without confirmation of a definitive cause of colic.

Duration of the ultrasonographic examination ranged from 12 min to 42 min (median 18 min). The attending clinician was at liberty to capture additional images or to investigate additional locations during their ultrasound examination, which has caused longer examination times in some instances. Images taken in additional locations were not recorded. The transrectal transducer examination was discontinued before completion in one case (left side of the abdomen) as it exceeded the length of time to complete the FLASH exam using the abdominal probe.

There was no statistically significant difference in detection of abdominal or thoracic free fluid and of the small intestine and its abnormalities, such as distention/liquid content, wall thickness and motility (Table 1). Although the colon wall was found to be thickened in 1.4% of the images captured with the abdominal transducer and in 5.6% of the images captured with the transrectal transducer, there was no statistically significant difference (Table 1). The detection of the left kidney was analyzed separately for the novices and experts and was found to be significantly different between the transducers for both novices and experts. The detection rate of the stomach was also significantly different between transducers with the stomach being seen in 88% of the images with the abdominal transducer, but only 63% of the images with the transrectal transducer. Detailed results can be found in Table 1. Due to the low identification rate of the stomach, comparative statistics between the two transducers for dilation of the stomach [identification of the stomach past the 15th ICS or its presence in more than 5 ICS (11)] was not performed.

**Table 1:** Identification of pathologies and organs using the abdominal and transrectal transducers.

Abdominal structure/Abnormality	Abdominal Transducer Identification rate in %	Transrectal Transducer Identification rate in %	*P*-value
Free fluid abdomen	27.78	29.17	1.0000
Free fluid thorax	1.42	8.57	0.1156
Duodenum seen	81.94	68.06	0.0824
Duodenum abnormal motility	8.47	6.12	0.25
Duodenum liquid content/distention	5.08	14.29	0.75
Other small intestine (not duodenum) (OSI) thickened wall	9.72	8.33	1.0000
OSI seen	95.83	94.44	1.0000
OSI abnormal motility	27.54	19.12	>0.9999
OSI liquid content/distention	34.78	41.18	>0.9999
Colon thickened wall	1.39	5.56	0.3662
L Kidney detected by ultrasonographers	91.67	37.50	0.0005 *
L Kidney detected by experts	81.25	22.92	<0.0001*
Stomach seen	87.50	62.50	0.0005 *

Statistically significant differences between transrectal and abdominal transducers in identifying organs and pathologies are indicated with an asterisk (*) OSI = Other small intestine, that was not identified as duodenum, the visualization of the kidney was analyzed for experts and ultrasonographers separately in consistency with the previous study (9).

Most horses had a BCS of 5 (*n* = 7/24) or 6 (*n* = 8/24), with only one horse having a BCS below 5 (BCS 2), and the remaining horses having BCS 7 (*n* = 5/24) and BCS 8 (*n* = 3/24). No statistically significant difference could be established in detection rate of abnormalities between the different BCS groups.

When comparing the effect of the haircoat score on both transducers, there was a statistically significant difference (values reported as median) between cases that had detectable free peritoneal fluid vs. cases that did not have detectable free peritoneal fluid (haircoat score 2 vs. 3, *p* = 0.0261) and ability to ultrasonographically identify the duodenum (haircoat score 2 vs. 3, *p* = 0.0265).

## 4 Discussion

To the authors’ knowledge this is the first clinical study to compare the incidence of detecting abnormalities using a transrectal and abdominal transducer in equine abdominal ultrasonography. The results suggest that the transrectal transducer can be used to identify many of the ultrasonographic abnormalities included in FLASH, except for conditions that require identification of the stomach and left kidney.

Transabdominal ultrasonography is a common diagnostic tool for assessment of equine colic (4–6). It provides valuable information to help differentiate surgical from non-surgical causes of colic and plays a role in deciding if cases should be referred for specialist care (4, 6). Farrell et al. demonstrated abnormal ultrasound findings along with 5 other values in horses with colic are correlated with survival, and that 76% of non-survivors have ultrasonographically detected abnormalities (12). Equine ultrasonographic examination of colic should include location, wall thickness, motility, diameter and content of the intestine, as well as the presence of abdominal or thoracic fluid (4, 8). Beccati et al. established statistically significant correlations between ultrasonographic abnormalities and definitive diagnoses in horses with colic. These included small intestinal strangulations with amotile small intestinal loops and thickened walls in conjunction with free peritoneal fluid, as well as a failure to visualize the left kidney with nephrosplenic entrapment (13).

In the current study, the FLASH protocol was used to evaluate horses with colic as it has been validated for use by operators with minimal ultrasonographic experience, and evidence suggests it can diagnose the most common causes of colic in the emergency setting (6). An abbreviated ultrasound protocol was chosen as it is deemed to be most applicable to first-opinion practice, even though it may have underestimated the severity of conditions in some horses, or exacerbated differences between the two transducers by reducing the imaging time and locations (2, 6).

**Figure 1:**
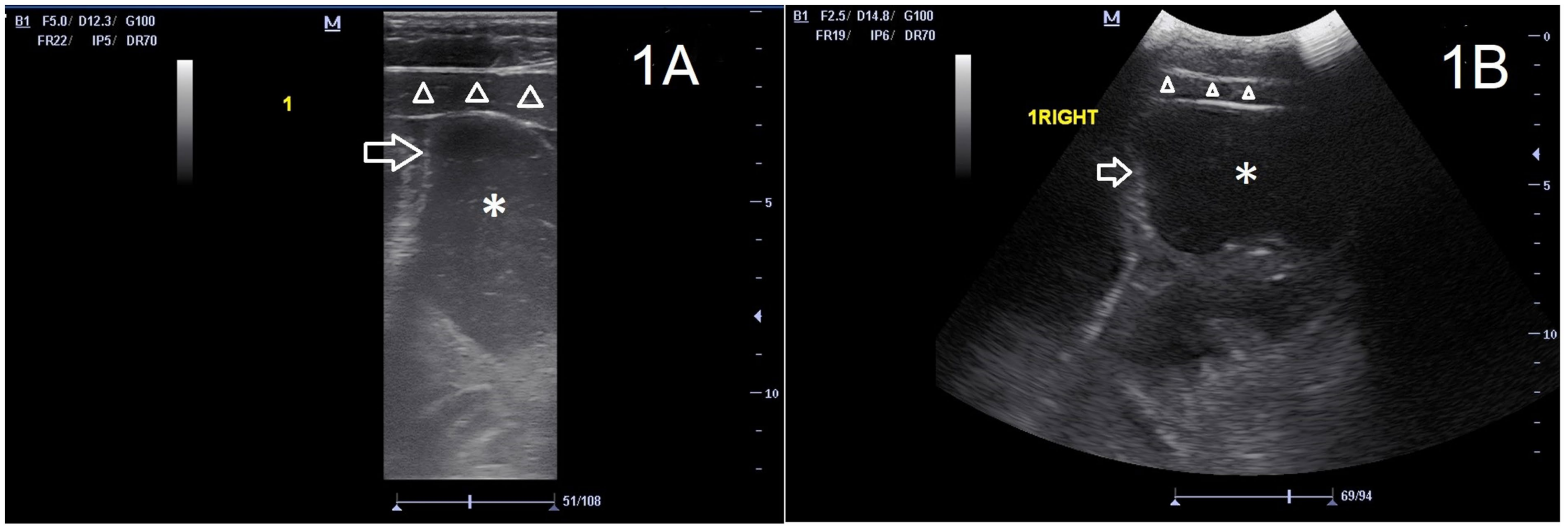
Images of the ventral abdomen (location 1) of the same horse, with a mildly distended loop of small intestine (asterisk) with wall of normal thickness (arrow). Ventral abdominal musculature (triangles) and subcutis are also visible in the near field. The left side of the image is cranial of the horse. **(A)** transrectal transducer, frequency 5 MHz, depth 12.3 cm. **(B)** abdominal transducer 2.5 MHz, depth 14.5 cm. Both transducers allow the ultrasonographer to identify the distention of the small intestine.

There was no statistically significant difference in detection of small intestines between the two transducers, with both identifying “other small intestine” (all small intestine that was not identified as duodenum) and duodenum in 94–98% of cases and 68–82% of cases. The presence of distended small intestinal loops [diameter > 7 cm (4)] can be associated with both nonsurgical lesions (e.g., ileus and smaller impactions of the ileum) and surgical lesions (e.g., strangulation of the small intestine). A recent study found that abnormal small intestinal findings in the ventral and left inguinal locations identified using the FLASH protocol were the best predictors for surgical intervention (14). Therefore, rapid diagnosis using a readily available transrectal probe and ultrasound machine under field conditions could lead to faster referral and improved survival rates. The transrectal transducer was consistently able to identify small intestinal loops in sufficient detail to appreciate abnormalities such as distention, increased wall thickness and absence of normal motility (Figure 1).

**Figure 2:**
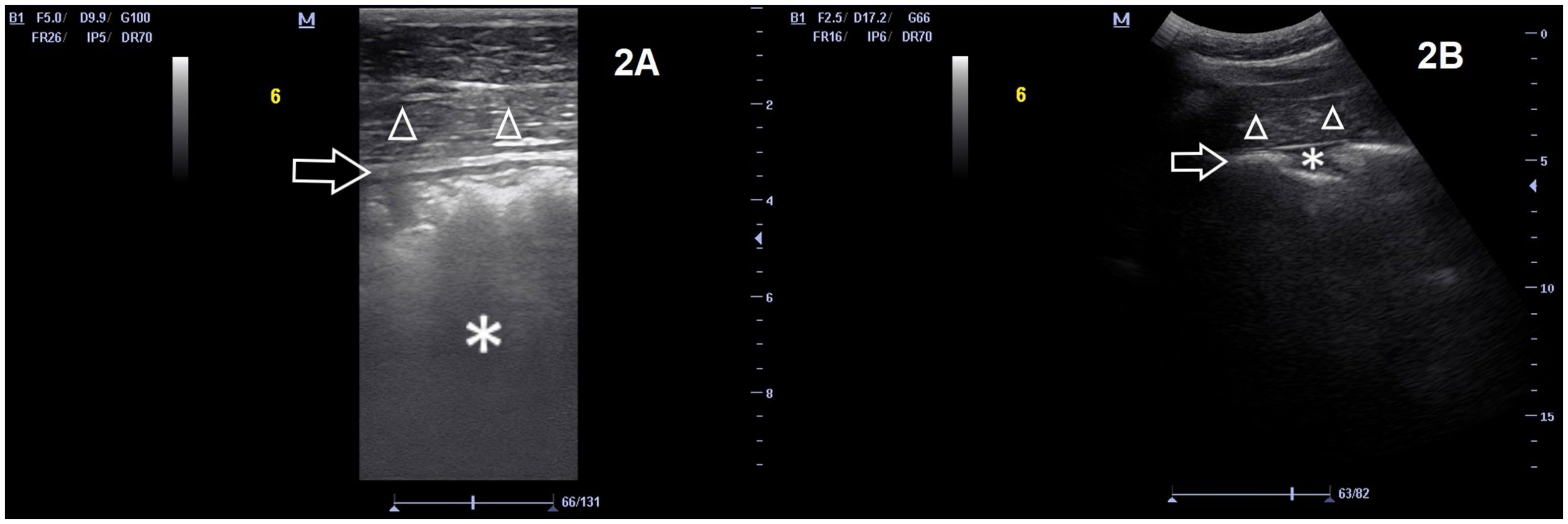
Images of the right lateral abdomen (location 6) of the same horse, with the numerous layers of the colon wall more distinct and visible in the near field (arrow). A small pocket of effusion is visible in **(B)** (asterisk). Body wall is also visible ventral to the colon (triangles). The left side of the image is cranial of the horse. **(A)** transrectal transducer, frequency 5 MHz, depth 9.9 cm. **(B)** abdominal transducer 2.5 MHz, depth 17.2 cm. Both images show a normal wall thickness, however, the magnification on the image taken with the rectal transducer allows an inexperienced ultrasonographer to measure the wall thickness with more ease.

Increased intestinal wall thickness can be associated with inflammation (colitis/enteritis) and strangulating lesions such as volvulus or torsion of the small and large intestines (4, 15–17). While abdominal ultrasound is not imperative to diagnose colon torsions, a study has found a wall thickness of the colon of > or = 9 mm to be highly specific (100%) and moderately sensitive (67%) (15). Strangulating lesions are treated surgically or lead to euthanasia of the patient, while enteritis and colitis are often managed with intensive medical treatment. Recognition of both these disease processes is important for early referral of the patient to a facility with the option of hospitalization and intensive care. A colonic wall thickness > 0.5 cm is regarded as pathological, while the cutoff for the small intestine lies at 0.2–0.35 cm (15, 16, 18–20). There was no statistically significant difference in detection of increased intestinal wall thickness. Nevertheless, a thickened colonic wall was seen in 4 instances with the transrectal transducer: one horse with a left dorsal displacement of the colon, two with colon impaction, and one with colitis, and only 1 case with a secondary impaction in combination with a right dorsal displacement with the abdominal transducer. This difference may be explained by the low-depth penetration but high near-field resolution of the transrectal probe, which at a lower depth settings, allows for an enlarged colon wall on the ultrasound image and emphasizes near-field structures relative to the transrectal transducer. Subtle changes may therefore be more obvious to the examiner using a linear probe than images captured using a lower resolution abdominal transducer. There was no difference in detection of changes in motility or content between transducers (Figure 2). An ultrasonographic examination with the transrectal probe therefore has similar ability compared to the abdominal probe in aiding the differentiation between a strangulating and an inflammatory lesion of the small and large intestines.

In accordance with a previous study performed by the same authors, the detection rate of the left kidney was significantly lower with the transrectal transducer (25%) compared to the abdominal transducer (85%), likely due to the kidney being positioned deep to the 5–8 cm-thickness equine spleen (8). With the maximum depth at which the transrectal transducer delivers images being 12 cm, a kidney located more than 10 cm from the skin surface and deep to the spleen would be difficult to visualize (9). Detection of the left kidney during FLASH is important since failure to image the kidney is associated with a diagnosis of left dorsal displacement of the large colon, although this finding is not definite (3, 4). In our study 4 horses were suspected to have a left dorsal displacement of the large colon. This was based on rectal palpation in conjunction with ultrasonographic findings detected with the abdominal probe, such as inability to image the spleen due to obscuration with gas. A diagnosis of nephrosplenic entrapment was made in one horse in which the colon could be felt in the nephrosplenic space on rectal palpation and where the dorsal aspect of the spleen was obliterated by gas on ultrasonography, as described in the literature (21). The majority of these were not detected with the transrectal transducer, making it unsuitable for assessment of this condition. Medical treatment in these cases included IV fluids, jogging, and administration of phenylephrine at the clinician’s discretion. Resolution of the left dorsal displacement or nephrosplenic entrapment was confirmed with rectal palpation, improvement of clinical signs, and in some cases repeat ultrasound.

The identification rate of the stomach using the transrectal transducer was lower than that with the abdominal transducer. This finding was consistent with the data of the previous study in which the stomach could be detected in 100% of horses using the abdominal probe, but in only 50% of cases using the transrectal transducer (9). Other equine studies report ultrasonographic variability in detecting the stomach of horses using an abdominal transducer. A study by Epstein et al. imaged the stomach in 7/9 ponies, Williams et al. excluded the stomach due to inconsistencies in ultrasonographically identifying it during their pilot work, while Freeman et al. state that the ability to ultrasonographically detect the stomach is inconsistent in normal horses (2, 17, 20). In the current study the detection rate of the stomach with the abdominal transducer was 87.5%. The relative inexperience of the veterinarian operating the ultrasound machine may also have contributed to the lower detection rate seen in this study compared to the previous study by the same authors. The number of cases with stomach distention was too low to allow statistical analysis, and a study with a larger number of cases is needed to identify any difference between transducers.

Air trapped between the hair of horses can reflect ultrasound waves, which is why many authors recommend clipping horses prior to performing ultrasonographic examinations (3, 16). In the current study isopropyl alcohol was liberally applied to the hair coat of all horses without clipping. However, to examine the influence of the length of the horses’ haircoat, horses were divided into four categories ranging from clipped to heavily coated. While there was no difference in detection rate of abnormalities seen, abdominal fluid detection and identification of the duodenum were seen more often in horses with a clipped or light coat with the transrectal transducer. Free fluid is most commonly detected at the lowest point of the abdomen. Often this is also where the horse’s haircoat is the longest. The transrectal transducer may have thus been more affected by the long haircoat than the abdominal transducer. However, this does not explain the influence of the haircoat on the ability to detect the duodenum. A possible explanation could be the limited field in which the duodenum can be viewed, making it more unlikely to be detected than other organs due to reduced ability to recognize surrounding structures with characteristic proximity such as the right kidney, right dorsal colon, and liver that help to distinguish the duodenum (22). A statistically significant difference between detection of the stomach in lighter coated horses with the abdominal transducer vs. the linear transducer could not be established. The number of horses with a light haircoat may not have been sufficient to reach statistical significance for this value. Further studies are warranted to establish a possible association between thickness of haircoat and ability to identify abnormal structures on ultrasound.

This study found BCS did not influence the ability to detect abnormalities on FLASH between transducers. Therefore, a cutoff value for BCS and detection of abnormalities using the transrectal transducer could not be determined. This was likely caused by the relatively homogeneous distribution of BCS in our study population with BCS 5 and 6 being overrepresented (63% of the horses fell within these two BCS scores).

A main limitation of this study was that severely painful horses were excluded due to the clinical need for immediate surgery without ultrasonographic examination. This was done to ensure animal welfare and a high standard of care for the horses. A second limitation was that experts graded cine-loops captured by veterinarians with limited experience. The presence of a winter haircoat on the horses examined may have impacted the results of the study, yet this mimics the circumstances of evaluating horses in the field during time-sensitive colic evaluations. The relative inexperience of the veterinarians may have resulted in suboptimal image quality and artificially lowered detection rates of abnormalities and limited the findings of this study. However, this also likely correlates with the experience of field veterinarians that do not frequently perform abdominal ultrasound. Even though the veterinary examiners had various years of previous experience, all of them had introduced abdominal ultrasound into their daily practice at the same time, and thus similar experience in abdominal ultrasound of horses. The veterinarian (H.H.) operating the transrectal transducer had previously participated in the first study. This may have enhanced the quality of cine-loops captured by this examiner in comparison to the veterinarians operating the abdominal transducer. Finally, there were relative low numbers in the subcategories of the large and small intestine (motility and content) which may have led to a type 2 error.

## 5 Conclusion

In conclusion, the transrectal transducer achieves comparable identification rates with the abdominal transducer for all abnormalities included in the FLASH protocol in horses presenting for colic, except those affecting the left kidney and stomach. These findings were consistent with the previously published data in healthy horses (9).

## Data availability statement

The original contributions presented in the study are included in the article/supplementary material, further inquiries can be directed to the corresponding author.

## Ethics statement

The animal studies were approved by University of Calgary Animal Care Committee. The studies were conducted in accordance with the local legislation and institutional requirements. Written informed consent was obtained from the owners for the participation of their animals in this study.

## Author contributions

HH: Conceptualization, Data curation, Investigation, Writing – original draft. AER: Conceptualization, Data curation, Investigation, Methodology, Supervision, Writing – review & editing. SRB: Conceptualization, Formal analysis, Supervision, Writing – review & editing. JYT: Conceptualization, Data curation, Investigation, Funding acquisition, Methodology, Project administration, Resources, Supervision, Writing – review & editing.

## Footnotes

^1^Walklate D. Heska Canada Limited (National Product Manager - Ultrasound and Advanced Imaging), Calgary, Canada (personal communication) 2021.

^2^Moore Equine Veterinary Center, Balzac, Alberta, Canada.
